# Protective Effects of Heat-Killed *Ruminococcus albus* against β-Amyloid-Induced Apoptosis on SH-SY5Y Cells

**DOI:** 10.4014/jmb.2308.08045

**Published:** 2023-10-31

**Authors:** Seungmoon Choo, Mirae An, Young-Hee Lim

**Affiliations:** 1 Department of Healthcare Sciences, Graduate School, Korea University, Seoul 02841, Republic of Korea; 2 Department of Integrated Biomedical and Life Sciences, Graduate School, Korea University, Seoul 02841, Republic of Korea; 3 School of Biosystems and Biomedical Sciences, College of Health Science, Korea University, Seoul 02841, Republic of Korea; 4 Department of Laboratory Medicine, Korea University Guro Hospital, Seoul, 08308, Republic of Korea

**Keywords:** *Ruminococcus albus*, β-amyloid, apoptosis, SH-SY5Y cells, neuroprotection

## Abstract

A high level of β-amyloid (Aβ) in the brains of patients with Alzheimer’s disease (AD) generates reactive oxygen species that induce neuronal death and DNA damage. The interaction between the gut microbiota and brain health has attracted attention in recent years. Heat-killed *Ruminococcus albus* (hkRA) reportedly protects neurons against damage induced by oxidative stress. However, whether hkRA can inhibit Aβ-induced apoptosis and thus alleviate AD remains unclear. Hence, we aimed to evaluate the protective effects of hkRA against Aβ-induced apoptosis on the human neuroblastoma SH-SY5Y cell. HkRA treatment (10^8^ cells/ml) significantly decreased the Aβ-induced cytotoxicity and DNA damage in the SH-SY5Y cells. It also showed a significant increase of the *bax/bcl-2* ratio in the Aβ-treated SH-SY5Y cells. Moreover, hkRA treatment stimulated the expression of antioxidation-related genes *HO-1*, *Nrf2*, and *PKC-δ* and increased the expression of brain-derived neurotrophic factor (*BDNF*). Meanwhile, it significantly decreased the activity of caspase-3 and protein expression of cleaved caspase-3 in the Aβ-treated SH-SY5Y cells. Additionally, the protein levels of mitochondrial and cytosolic cytochrome c increased and decreased, respectively, in the cells. These results suggest that hkRA protects human neuroblastoma cells from Aβ-induced apoptosis and oxidative stress. Thus, hkRA may be developed into a health-promoting paraprobiotic (the inactivated microbial cells of probiotics) for patients with AD.

## Introduction

Dementia is an aging-related disease that has been actively studied because of the severe global impact of societal aging. This disease has several types, including Alzheimer’s disease (AD), alcoholic dementia, vascular dementia, and Parkinson’s disease (PD). Among the types of dementia, AD has become an important medical and social problem because it is the most common among elderly people. β-Amyloid (Aβ) is a major component of amyloid plaques that cause AD [1]. It is derived from amyloid precursor protein, which is degraded by beta- and gamma-secretases [2]. Aβ accumulation in the brain stimulates neuronal damage, destroys synapses, and adversely affects cognitive function [3-5]. Aβ-induced brain neuronal cytotoxicity is closely related to oxidative stress caused by intracellular increase in reactive oxygen species (ROS) levels, and oxidative damage caused by ROS overproduction is a major cause of degenerative brain disease [6, 7]. Antioxidants inhibit Aβ-induced apoptosis, which prevents brain diseases, such as AD [8, 9]. However, the mechanisms by which Aβ affects brain cells remain to be elucidated. Aβ, which is composed of 38–43 amino acids, is neurotoxic in vitro and in vivo. Synthetic Aβ (1−40) and Aβ (25−35) are also neurotoxic [10]. In particular, Aβ (25−35), the most toxic peptide fragment, has been universally used to induce AD.

The bidirectional communication through the gut–brain axis affects brain cognition, emotion, and behavior [11-13]. The gut–brain axis also interacts with the nervous, endocrine, and immune systems through connections between intestinal microbes, metabolites, and the brain. Gut dysbiosis is a known, important cause in various neuropsychiatric disorders, such as attention deficit hyperactivity disorder, learning disabilities, panic disorders, AD, and PD [14]. The intestinal microbiome plays an important role in this interaction [15, 16].

Until recently, the drug treatment for AD was based on the cholinergic hypothesis because acetylcholine has been thought to promote memory and learning. This therapeutic approach focuses on the inhibition of acetylcholinesterase by FDA-approved reversible acetylcholinesterase inhibitors, such as donepezil, galantamine, and rivastigmine, and memantine, a N-methyl-D-aspartate receptor antagonist [17]. AD is also alleviated by anti-inflammatory drugs, estrogen, antioxidants, Aβ protein deposition, and aggregation inhibitors [18]. However, none of these therapeutics have been used for the complete treatment of AD.

Recently, clinical trials have shown that probiotics are promising for AD prevention [19, 20]. Live and inactivated probiotics exert similar health benefits [21-23]. Nevertheless, inactivated probiotics (known as paraprobiotics) have some advantages over live probiotics, such as no risk of infection in compromised individuals, stability, and ease of production and handling (transportation and storage). Heat-killed probiotics are currently receiving attention for their health-promoting effects, but only limited information is available [24, 25]. *Ruminococcus albus* is a gram-positive intestinal bacterium that is primarily cellulolytic. In our previous study, we found that substances secreted from heat-killed *R. albus* (hkRA)-treated intestinal epithelial Caco-2 cells increase the proliferation of neuronal SH-SY5Y cells and protect them against H_2_O_2_-induced oxidative stress by activating the expression of brain-derived neurotrophic factor (BDNF), serum response factor, and cyclin-dependent kinase 2 [26]. In addition, hkRA protects neurons from oxidative damage induced by sodium arsenate in rats. Considering the neuronal cell death in AD caused by oxidative stress and Aβ protein accumulation [2, 6] and the protective potency of hkRA on oxidative stress-induced neuronal damage, we hypothesized that hkRA itself and not its metabolites can alleviate AD by inhibiting Aβ-induced neurotoxicity. In this study, we aimed to investigate the direct protective effects of hkRA against Aβ-induced apoptosis not through the gut-brain axis, thereby preventing DNA damage, and elucidate the underlying mechanisms in SH-SY5Y cells.

## Materials and Methods

### Preparation of hkRA

*Ruminococcus albus* KCTC 15045 was obtained from the Korean Collection Type Culture (Republic of Korea). The bacterium was cultured under anaerobic conditions in modified DSMZ 453 medium (Leibniz Institute, Germany) at 37°C. *R. albus* was harvested by centrifugation at 3,000 ×*g* for 5 min and washed with phosphate-buffered saline (PBS). After centrifugation, the pelleted cells were resuspended in PBS by gentle vortexing and were heat killed at 100°C for 10 min and then stored in a ‒80°C deep freezer until further use.

### Preparation of Aβ_25–35_ Aggregates

Aβ_25–35_ (Sigma-Aldrich, USA) (1 mM) in distilled water stored at ‒20°C until use. For aggregation of Aβ_25–35_ to increase cytotoxicity, it was incubated in a 37°C water bath for 72 h [27]. In all experiments, Aβ_25–35_ was treated to the final concentration of 10 μM.

### Cell Culture

The human neuroblastoma SH-SY5Y cell line was purchased from the Korean Cell Line Bank (Republic of Korea ). SH-SY5Y cells were cultured in DMEM supplemented with high glucose containing 10% fetal bovine serum (FBS) and penicillin–streptomycin (100 U/ml each) (HyClone, USA) at 37°C in 5% CO_2_. All experiments were performed 24 h after seeding the cells onto the plates. SH-SY5Y cells were pretreated with various concentrations (10^6^, 10^7^, and 10^8^ cells/ml) of hkRA for 30 min and then exposed to 10 μM Aβ_25–35_ dissolved in distilled water for 24 h. Only cell passages under 30 were used in all experiments.

### Cell Viability

SH-SY5Y cells were plated at 1 × 10^5^ cells/well in a 96-well cell culture plate and incubated at 37°C in 5% CO_2_ and 95% air. After 24 h, the cells were treated with various concentrations of hkRA (10^2^–10^8^ cells/ml) and incubated at 37°C in 5% CO_2_ for 24 h. The effects of hkRA and Aβ_25–35_ (10 μM) on SH-SY5Y cell viability were measured as follows: Cells were serum-starved overnight and pretreated with hkRA for 30 min and then treated with Aβ_25–35_ (10 μM) for 24 h. Cell viability was assessed using the 3-[4,5-dimethylthiazol-2-yl]-2,5-diphenyltetrazolium bromide (MTT) assay in accordance with the manufacturer’s protocol (Amresco, USA). Cell viability (%) was calculated as a percentage relative to the negative control (NC) without hkRA treatment.

### Soft Agar Colony Formation Assay

A soft agar colony formation assay (clonogenic assay) was performed to evaluate the effect of hkRA pretreatment on the proliferation of Aβ_25–35_-treated SH-SY5Y cells. For the base layer, a mixture composed of 0.5%agar, DMEM, and 10% FBS was prepared. In a 6-well plate, 1 ml of the mixture was added to each well and allowed to solidify for 5 min. For the top layer, 5 × 10^3^ cells/well were mixed with 0.35% low-melting agarose (Sigma-Aldrich) and Aβ_25–35_ (10 μM). Various concentrations (10^6^, 10^7^, and 10^8^ cells/ml) of hkRA were also added to the top-layer agar. The cells were incubated at 37°C in 5% CO_2_ for 2 weeks. The plates were fixed with formaldehyde and then stained with crystal violet for 30 min.

### Comet Assay

DNA damage was evaluated using the comet assay, which involves measuring the migration distance of the nuclei through electrophoresis of the DNA of damaged cells. SH-SY5Y cells were harvested by centrifugation at 400 ×*g* for 5 min. The cells were mixed with 0.75% low-melting point agarose and immediately spread on frosted microscope slides precoated with 0.75% normal agarose. The slides were covered with coverslips and incubated to allow gel solidification for 10 min at 4°C. Then, the slides were immersed in ice-cold lysis solution (2.5 M NaCl, 100 mM EDTA, 10 mM Tris, and 1% Triton X-100, pH 10) for 1 h at 4°C. After lysis, the slides were placed in electrophoresis buffer overnight and then electrophoresed at 60 V for 20 min. After electrophoresis, the slides were neutralized with 0.4 M Tris, pH 7.5. The gels were stained with propidium iodide (Sigma-Aldrich) and dried overnight at room temperature. Images were obtained using a confocal microscope (C1 PLUS; Nikon, Japan). The tail moment (tail length × DNA in tail (%)) was calculated using CaspLab software (version 1.2.3. beta 2)(CASPlab, Wroclaw, Poland).

### Quantitative Real-Time Polymerase Chain Reaction (qPCR)

Total RNA was extracted using AccuZol Total RNA Extraction Reagent (Bioneer, Korea) in accordance with the manufacturer’s protocol. The PCR primers were purchased from Bioneer (Table 1). The PCR conditions were as follows: 95°C for 10 min, followed by 40 cycles at 95°C for 15 s, 60°C for 15 s, and 72°C for 20 s. Glyceraldehyde 3-phosphate dehydrogenase (*GAPDH*) was used as the internal control gene. Normalized expression change was expressed as 2^–ΔΔCt^ (The control *GAPDH* was set to 1).

### Caspase-3 Activity

Caspase-3 activity was measured using a caspase-3 colorimetric assay kit (BioVision, USA) in accordance with the manufacturer’s instructions. Briefly, SH-SY5Y cells were lysed in a caspase-3 sample lysis buffer and then incubated on ice for 10 min. After 10 min, the lysate was centrifuged at 10,000 ×*g* at 4°C for 1 min. The supernatant was incubated with DEVD-pNA substrate (200 μM final concentration) at 37°C for 1 h, and the absorbance was obtained at 405 nm using the SpectraMax 340PC384 plate reader (Molecular Devices, USA).

### Western Blot

Proteins were extracted using the Pro-prep Protein Extract solution (Intron, Republic of Korea) in accordance with the manufacturer’s protocol. Mitochondrial and cytosolic protein fractions were prepared using a mitochondria/cytosol fractionation kit (Biovision). In brief, cells were incubated in the Pro-prep solution at ‒20°C for 30 min and then centrifuged at 13,000 ×*g* for 5 min at 4°C. The supernatants were collected, and the protein concentrations were measured using Bradford assay. Equal amounts (30 μg) of proteins for each sample were separated through 10% SDS-PAGE and then transferred onto polyvinylidene difluoride membranes (Millipore, USA) using a Trans-Blot semi-dry transfer cell (Bio-Rad, USA). The membranes were blocked with 5% (w/v) skim milk (Bio-Rad) in PBS-T (PBS containing 0.05% Tween-20) at room temperature for 1 h, washed three times with PBS-T for 10 min, and then incubated with primary antibodies against β-actin (1:5,000 dilution, MA5-15739; Thermo Fisher Scientific), caspase-3 p11 (1:500 dilution, sc-271759; Santa Cruz Biotechnology, USA), and cytochrome c (1:500 dilution, sc-13156; Santa Cruz Biotechnology) for 1 h at room temperature. After being washed three times with PBS-T for 10 min each, the membranes were incubated with goat anti-mouse IgG (H+L) horseradish peroxidase-conjugated secondary antibodies (1: 20,000 dilution; NCI1430KR, Thermo Fisher Scientific) for anti-β-actin, anti-caspase-3 p11, and anti-cytochrome C for 1 h at room temperature. Proteins were detected using the SuperSignal West Femto Maximum Sensitivity Substrate Kit (Thermo Fisher Scientific). Blot images were analyzed using the FluorChem E imaging system (ProteinSimple, USA). Protein density was quantified using ImageJ software (Sofomic, Barcelona, Spain).

### Statistical Analysis

All statistical analyses were performed using one-way analysis of variance (ANOVA) followed by Tukey’s honestly significant difference (HSD) test using SPSS version 24.0. Results are expressed as the mean ± SD of three independent experiments performed in triplicate. A *p*-value of less than 0.05 was considered statistically significant.

## Results and Discussion

### Effects of hkRA Pretreatment on the Viability of Aβ_25–35_-Treated SH-SY5Y Cells

**Fig. 1:**
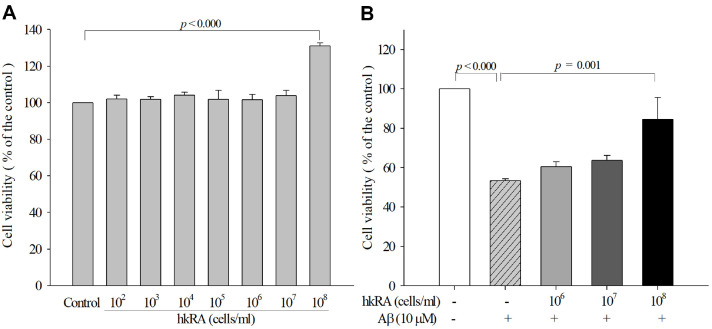
Effect of heat-killed *Ruminococcus albus* (hkRA) on the viability of SH-SY5Y cells. SH-SY5Y cells were treated with various concentrations of hkRA (10^2^, 10^3^, 10^4^, 10^5^, 10^6^, 10^7^, and 10^8^ cells/ml) for 24 h, and the cytotoxic effect of hkRA was measured using the 3-[4,5-dimethylthiazol-2-yl]-2,5-diphenyltetrazolium bromide (MTT) assay (**A**). SH-SY5Y cells were pretreated with hkRA at concentrations of 10^6^, 10^7^, and 10^8^ cells/ml. After 30 min, the cells were treated with β- amyloid 25–35 (Aβ_25–35_) for another 24 h, and cell viability was measured using the MTT assay (**B**). Data are expressed as the mean ± SD of three independent experiments in triplicate. One-way ANOVA/Tukey’s honestly significant difference analysis was performed. ns; not significant compared with the control.

Cell viability was not significantly different in all hkRA-treated SH-SY5Y cells, except for the 10^8^ cells/ml of hkRA-treated cells. The hkRA concentration of 10^8^ cells/ml significantly increased the viability of the SH-SY5Y cells by 130.97% ± 1.66% compared with the NC (100%) (Fig. 1A). Therefore, hkRA at concentrations of 10^6^, 10^7^, and 10^8^ cells/ml was used in subsequent experiments in this study. The viability of the SH-SY5Y cells treated only with Aβ_25–35_ alone significantly decreased by 53.31% ± 0.94% compared with that of the NC (Fig. 1B). Although pretreatment with 10^8^ cells/ml hkRA only significantly (*p* = 0.001) protected the cells against Aβ_25–35_ toxicity, pretreatment with 10^6^ or 10^7^ cells/ml hkRA also increased cell viability.

Aβ_25–35_ induces neuronal apoptosis and is used as a substitute for Aβ_1–42_ because it immediately forms a β-pleated sheet structure similar to Aβ_1–42_ and has high toxicity [27]. In addition, the 25–35 region of Aβ contributes to the aggregation of amyloid fibrils and neurotoxicity in peptides. We found that Aβ_25–35_ was highly toxic to the SH-SY5Y cells. Without Aβ_25–35_ treatment, a significant difference in viability was not found between the cells pretreated with 10^2^‒10^7^ cells/ml hkRA and PBS. Moreover, the cells pretreated with 10^8^ cells/ml hkRA showed higher viability than those treated with the control, confirming the safety of hkRA at the concentrations used in this study. Treatment with Aβ_25–35_ significantly decreased the viability of the cells, and although this effect was not significant, the viability in the hkRA pre-treated-cells decreased as well compared with the NC. Nevertheless, pretreatment with hkRA, especially 10^8^ cells/ml hkRA, showed a significant protective effect against the Aβ_25–35_-induced cytotoxicity.

### Effect of hkRA Pretreatment on the Proliferation of Aβ_25–35_-Treated SH-SY5Y Cells

**Fig. 2:**
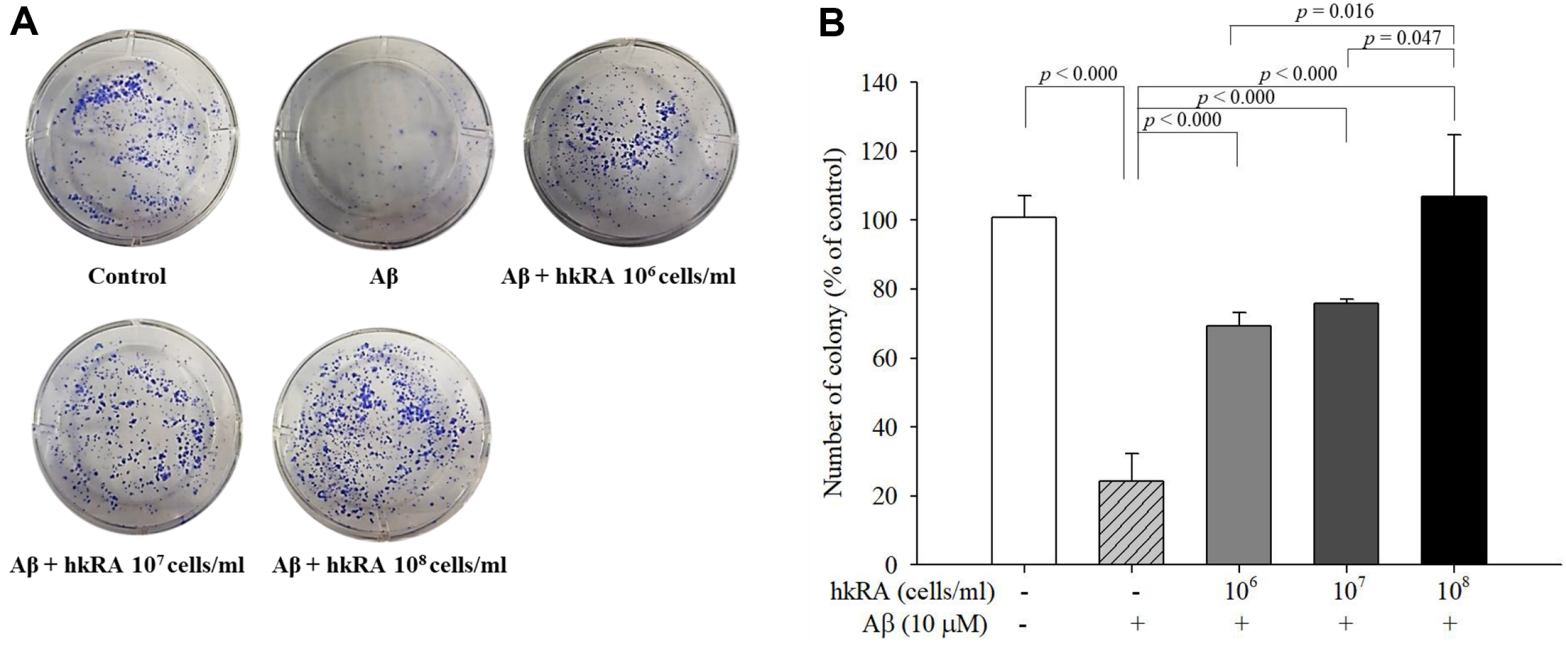
Proliferation of SH-SY5Y cells pretreated with heat-killed *Ruminococcus albus* (hkRA) before β-amyloid (Aβ) treatment. Representative colony formation images of hkRA-pretreated SH-SY5Y on soft agar plates (**A**) and the colonies were counted (**B**). Data are expressed as the mean ± SD of three independent experiments in triplicate. One-way ANOVA/Tukey’s honestly significant difference analysis was performed.

The effect of hkRA pretreatment on the proliferation of Aβ_25–35_-treated SH-SY5Y cells was evaluated through soft agar colony formation assay. The colony formation of the Aβ_25–35_ only-treated cells (24.30% ± 7.87%) was significantly lower than that of the cells treated with the control (100%) (Fig. 2). Pretreatment with hkRA showed a significant dose-dependent increase in the colony formation of the Aβ_25–35_-treated SH-SY5Y cells. The results suggested that hkRA protected the proliferation of cells under Aβ_25–35_ treatment.

### Neuroprotective Effect of hkRA on SH-SY5Y Cells Against Aβ_25–35_-Induced DNA Damage

**Fig. 3:**
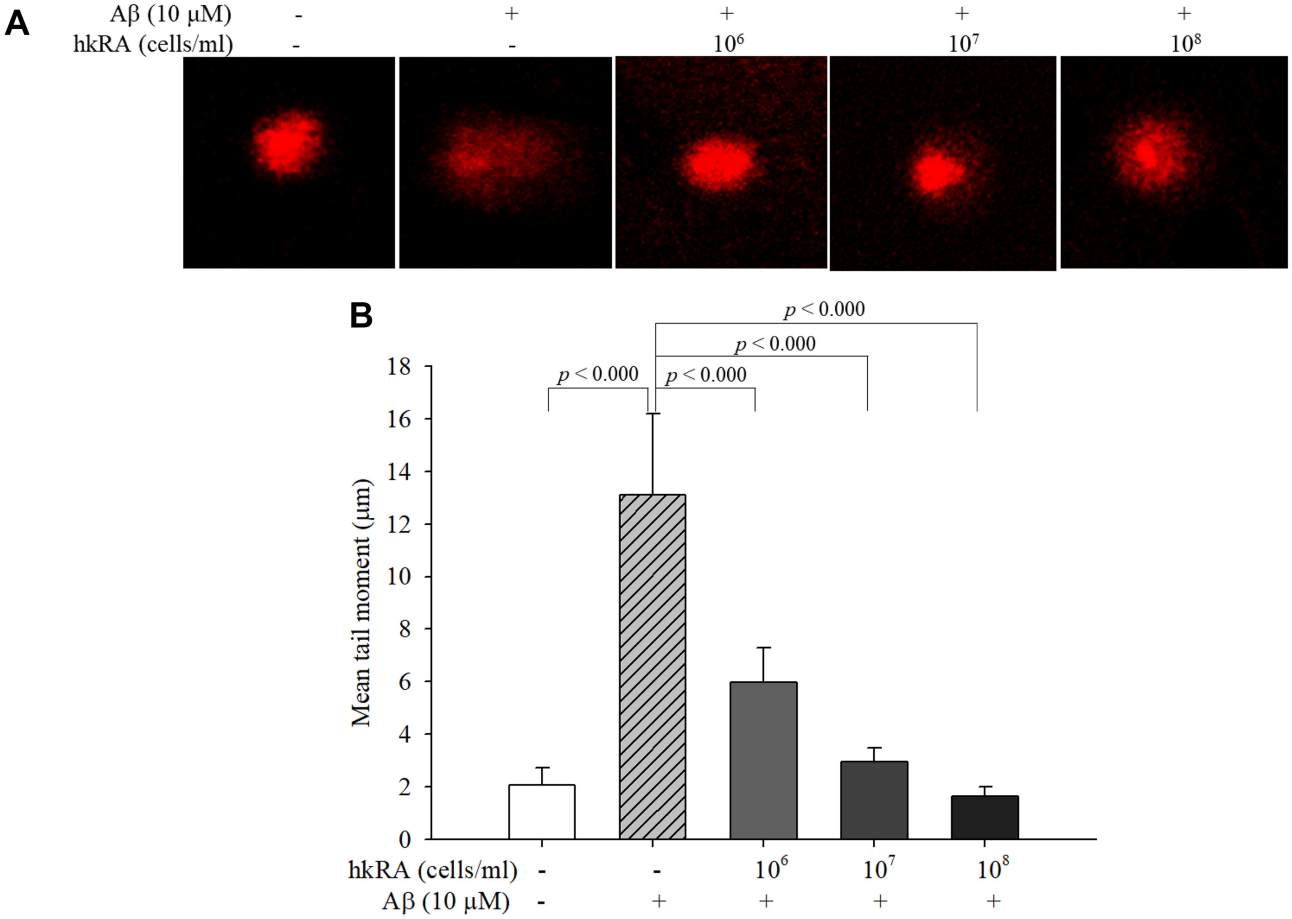
Effect of heat-killed *Ruminococcus albus* (hkRA) on β-amyloid 25–35 (Aβ_25–35_)-induced DNA damage in SH-SY5Y cells. DNA damage was measured using comet assay, and propidium iodide staining images were obtained by confocal microscopy (Scale bar: 5 μm) (**A**). Migration distance was measured using CaspLab software (version 1.2.3. beta 2), and the mean tail moment was calculated (**B**). Data are shown as the means±SD of three independent experiments in triplicate. One-way ANOVA/Tukey’s honestly significant difference analysis was performed.

Aβ_25–35_-induced DNA damage was investigated by a comet assay. The migration distances of the cells treated with the control and Aβ_25–35_ alone were 2.08 ± 0.64 and 13.10 ± 3.09 μm, respectively, indicating that Aβ_25–35_ treatment caused significant DNA damage to the SH-SY5Y cells. By contrast, the migration distances of the cells pretreated with 10^6^, 10^7^, and 10^8^ cells/ml hkRA were 5.98 ± 1.32, 2.94 ± 0.52, and 1.63 ± 0.36 μm, respectively (Fig. 3). The results showed that hkRA significantly reduced Aβ_25–35_-induced DNA damage in the SH-SY5Y cells.

### Effect of hkRA on the Expression of Apoptosis- and Antioxidation-Related Genes in Aβ_25–35_-Treated SH-SY5Y Cells

**Fig. 4:**
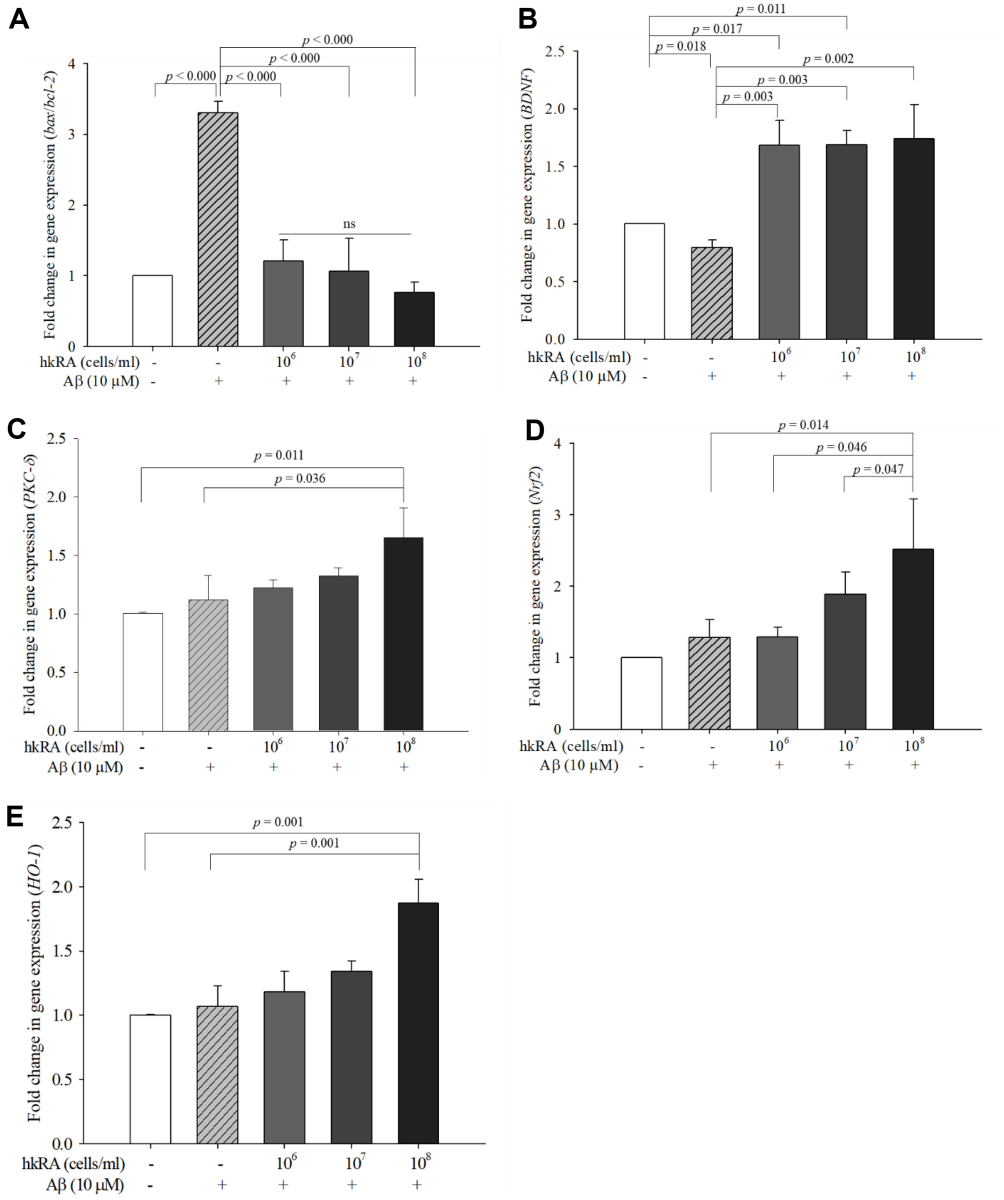
Effects of heat-killed *Ruminococcus albus* (hkRA) on the expression of genes related to neuroprotection in Aβ_25–35_-treated SH-SY5Y cells. The cells were pretreated with various concentrations of hkRA in Aβ_25–35_-treated SHSY5Y cells for 24 h. Expression levels of *bax/bcl-2* (**A**) *BDNF* (**B**) *PKC-δ* (**C**) *Nrf2* (**D**), and *HO-1* (**E**) in Aβ_25–35_-treated SH-SY5Y cells. Data are shown as the means±SD of three independent experiments in triplicate. One-way ANOVA/Tukey’s honestly significant difference analysis was performed.

The degree of apoptosis was determined by calculating the expression ratios between *bax* and *bcl-2*, a pro-apoptotic gene and an anti-apoptotic gene, respectively [28]. The expression ratio of *bax/bcl-2* significantly (*p* < 0.000) increased by 3.3-fold in the cells treated only with Aβ_25–35_ compared with the NC (Fig. 4A). However, this ratio decreased by 0.37-, 0.32-, and 0.23-fold in the cells pretreated with 10^6^, 10^7^, and 10^8^ cells/ml hkRA, respectively, compared with the cells treated with Aβ_25–35_ alone. Aside from markedly decreasing the distance of DNA fragment migration, hkRA pretreatment also decreased the *bax/bcl-2* ratio, confirming its protective effect against Aβ_25–35_-induced apoptosis in the SH-SY5Y cells.

The expression level of *BDNF* was significantly lower in the cells treated only with Aβ_25–35_ than in the NC cells; however, it was significantly higher in the cells pretreated with all concentrations of hkRA than in those treated only with Aβ_25–35_ (Fig. 4B). Interestingly, the expression of *BDNF* was significantly higher in the cells pretreated with all concentrations of hkRA than in those treated with the control. BDNF is a brain growth hormone that stimulates the growth and division of new neurons and aids in their survival [29]. BDNF levels decrease with aging, accelerating brain damage [30]. Thus, BDNF may be a key factor in delaying brain aging while also being an important target in improving degenerative brain diseases. Pretreatment with hkRA notably increased *BDNF* expression in the Aβ_25–35_-treated SH-SY5Y cells, suggesting that hkRA could be a neuroprotective substance.

Nerve damage can also be induced by ROS in apoptotic cells stimulated by Aβ [6]. Therefore, the expression of genes related to ROS inhibition may also be an important indicator of neuroprotective effects in Aβ-treated cells. Transcription factor Nrf2 (nuclear factor erythroid 2-related factor 2) is a major regulator of the expression of antioxidant proteins that maintain intracellular antioxidant activity and performs an important function in maintaining intracellular redox homeostasis. Nrf2 induces the transcription of various cytoprotective genes that prevent the deleterious effects of oxidative stress and toxicants. Heme oxygenase-1 (HO-1) is a typical antioxidant enzyme that responds to oxidative stress and is expressed through the PKC-δ/Nrf2/ARE signaling pathway in SH-SY5Y cells [7, 31]. In this study, the expression levels of antioxidation-related genes *PKC-δ*, *Nrf-2*, and *HO-1* were significantly higher in the cells pretreated with 10^8^ cells/ml hkRA than in those treated with Aβ_25–35_ alone (Fig. 4C-4E). This result suggested that hkRA showed a neuroprotective effect against oxidative stress by activating the PKC-δ/Nrf2/ARE signaling pathway in SH-SY5Y cells.

### Inhibitory Effects of hkRA on Mitochondrial Cytochrome c Release and Caspase-3 Activity in Aβ_25–35_-Treated SH-SY5Y Cells

**Fig. 5:**
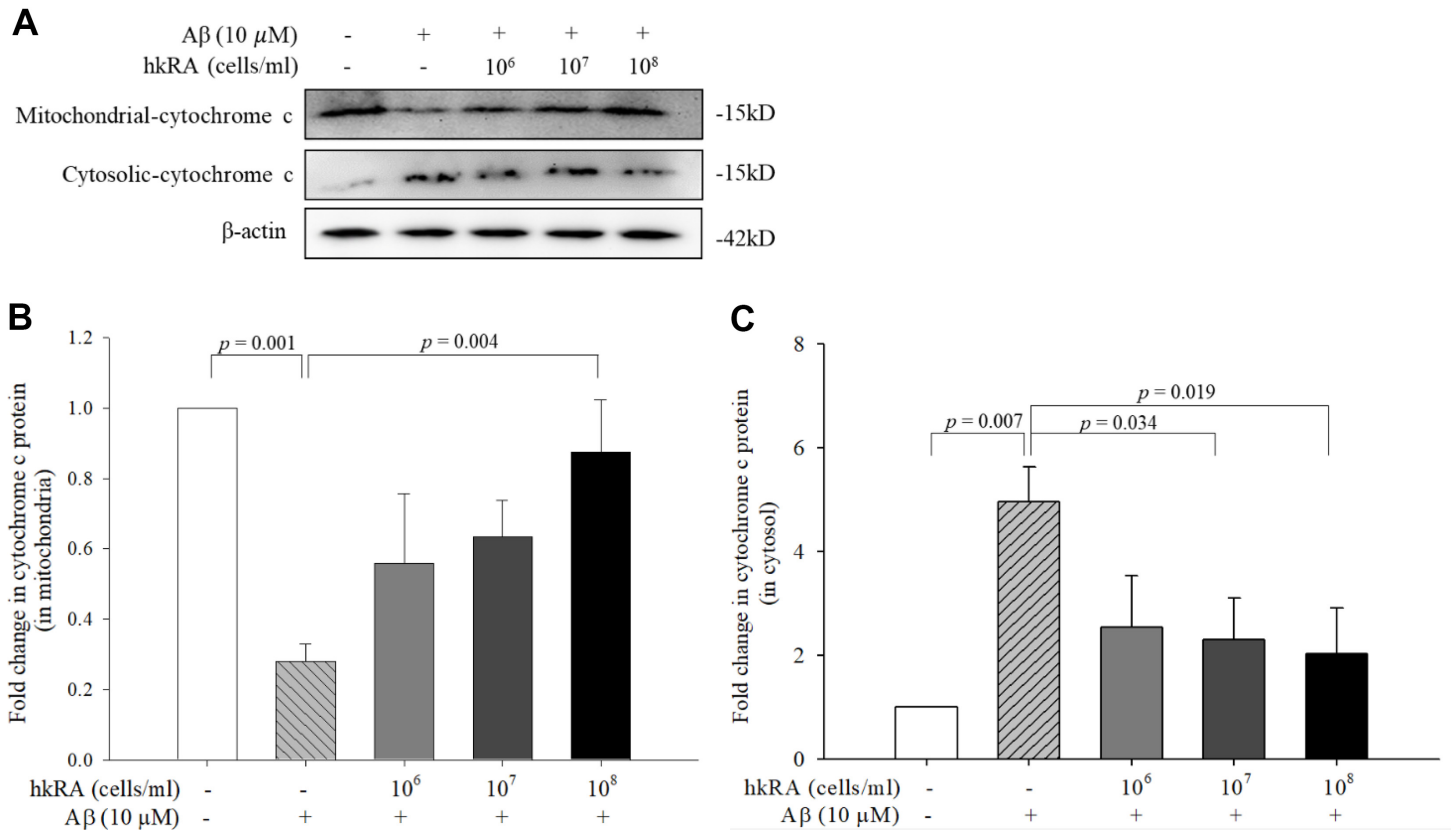
Inhibitory effect of heat-killed *Ruminococcus albus* (hkRA) on cytochrome c release in Aβ_25–35_-treated SH-SY5Y cells. Protein levels of cytochrome c were determined using western blot (**A**) and cytochrome c levels were quantified in the mitochondria (**B**) and cytosol (**C**). Data are expressed as the means±SD of three independent experiments. One-way ANOVA/Tukey’s honestly significant difference analysis was performed.

Cytochrome c release was measured through western blot to investigate the inhibitory effect of hkRA on cytochrome c release from the mitochondria to the cytoplasm in the Aβ_25–35_-treated SH-SY5Y cells (Fig. 5A). In the cells treated with Aβ_25–35_ alone, the protein level of cytochrome c in the mitochondria significantly decreased by 0.27-fold compared with the NC, but it significantly increased by 3.13-fold in the cells pretreated with 10^8^ cells/ml hkRA compared with the Aβ_25–35_ only-treated cells, and even recovered to 87.41% ± 0.15% that of the control (Fig. 5B). Meanwhile, the amount of cytochrome c released from the mitochondria to the cytoplasm significantly increased by 4.96-fold in the cells treated only with Aβ_25–35_ compared with the NC; however, it gradually decreased by 0.51-, 0.46-, and 0.41-fold in the cells pretreated with 10^6^, 10^7^, and 10^8^ cells/ml hkRA, respectively, compared with the cells treated with Aβ_25–35_ alone (Fig. 5C). The hkRA-treated cells showed significantly lower levels of released cytochrome c than the Aβ_25–35_ only-treated cells, indicating that hkRA pretreatment protected the SH-SY5Y cells from Aβ_25–35_-induced apoptosis by inhibiting the release of cytochrome c from the mitochondria to the cytoplasm.

**Fig. 6:**
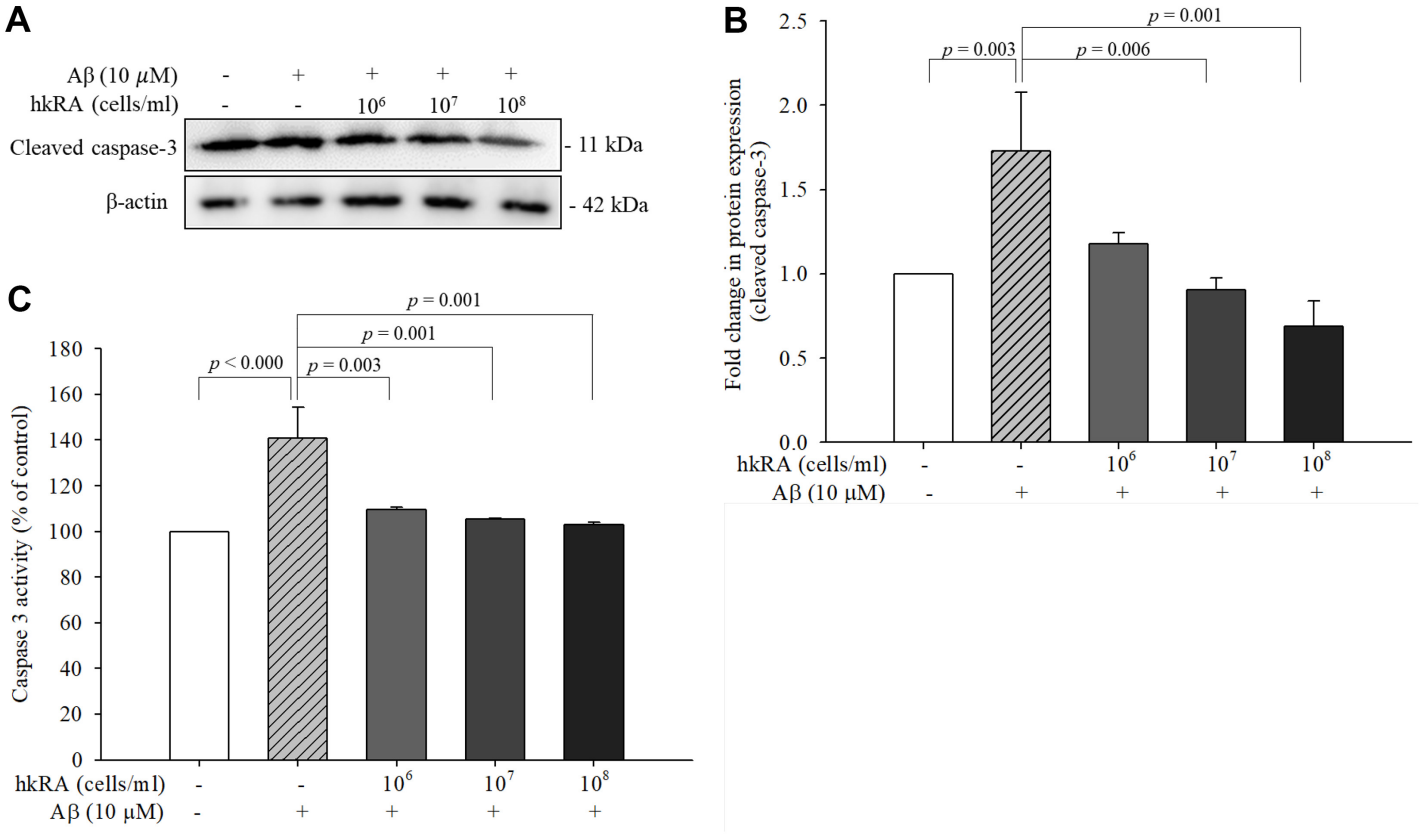
Effects of heat-killed *Ruminococcus albus* (hkRA) on the protein expression of cleaved caspase-3 and activity of caspase in Aβ_25–35_-treated SH-SY5Y cells. Protein expression of cleaved caspase-3 was determined using western blot (**A**) and quantified (**B**). Caspase activity was measured using a colorimetric assay (**C**). Data are shown as the means±SD of three independent experiments. One-way ANOVA/Tukey’s honestly significant difference analysis was performed.

The activity of caspase-3 and expression of cleaved caspase-3 were measured to evaluate the inhibitory effect of hkRA on Aβ_25–35_-induced apoptosis. The expression of cleaved caspase-3 significantly increased by 1.73-fold in the cells treated only with Aβ_25–35_ compared with the cells treated with the control; however, it significantly decreased by 0.52- and 0.40-fold in the cells pretreated with 10^7^ and 10^8^ cells/ml hkRA, respectively, compared with the Aβ_25–35_ only-treated cells (Fig. 6A and 6B). Caspase-3 activity significantly increased by 140.78% ± 13.52%in the Aβ_25–35_ only-treated cells compared with the NC. In contrast, it significantly reduced in the cells treated with all hkRA concentrations compared to the Aβ_25–35_ only- treated cells (Fig. 6C). These results showed that hkRA inhibited Aβ_25–35_-induced caspase-3 activity and thus may prevent Aβ_25–35_-induced apoptosis in the SH-SY5Y cells.

Oxidative stress and mitochondrial dysfunction are important causes of Aβ-mediated neurotoxicity [32]. Aβ accumulates in the mitochondria of AD patients’ brains, which reveal mitochondrial structural abnormalities [33], and results in the release of cytochrome c from the mitochondria to the cytoplasm [34]. Cytochrome c is released from the mitochondrial inner membrane and binds to Apaf-1 (apoptosis protease-activating factor), which then recruits procaspase-9 to form an apoptosome that cleaves procaspase-9 to activate caspase-9. Activated caspase-9 stimulates caspase-3, a marker of apoptosis. The level of cleaved caspase-3, a terminal enzyme in apoptosis, is significantly correlated with cancer progression [35]. The level of cleaved caspase-3 decreases as amyloid-β-induced neuronal damage is ameliorated by the overexpression of fibroblast growth factor 13 [36]. In the present study, hkRA pretreatment significantly reduced cytosolic cytochrome c level in the Aβ_25–35_-treated SH-SY5Y cells. In addition, it significantly reduced the activity of caspase-3 and the protein expression of cleaved caspase-3 in the Aβ_25–35_-treated SH-SY5Y cells. These results indicated that hkRA protected the cells from Aβ_25–35_-induced apoptosis by decreasing cytochrome c release from the mitochondria to the cytoplasm, which decreased the generation of cleaved caspase-3, the active form of caspase that plays an important role in apoptosis [37].

*R. albus*, an important cellulolytic bacterium in the intestine, is a next-generation probiotic, and healthy individuals possess higher loads of *R. albus* than patients with Crohn’s disease or immunodeficiency disorders [38, 39]. hkRA has health-promoting effects similar to those of live bacterial cells [26]. In the present study, hkRA pretreatment protected neurons from Aβ-induced neurotoxicity, suggesting that hkRA may be developed as a paraprobiotic for patients with AD. Additionally, further extensive study on the identification of the active component(s) involved in the protection against neuronal damage in hkRA is necessary.

In conclusion, hkRA prevents neuronal damage from Aβ_25–35_-induced oxidative stress and protects neurons from Aβ_25–35_-induced apoptosis. hkRA also upregulates the expression of *BDNF*, a major factor in the protection and restoration of neurons. Thus, based on the results obtained from this in vitro study using the SH-SY5Y cell model, further in vivo studies are needed to justify if hkRA passes directly through the blood-brain barrier, and its improvement effect on Aβ_25–35_-induced neuronal diseases, such as AD, by protecting neurons from damage and promoting their growth.

## Figures and Tables

**Table 1:** Primer sequences used for qPCR.

Gene	Forward (5'–3')	Reverse (5'–3')
*GAPDH*	GAGTCAACGGATTTGGTCGT	GACAAGCTTCCCGTTCTCAG
*Bax*	GTGGTTGCCCTCTTCTACTTTGC	GAGGACTCCAGCCACAAAGATG
*Bcl-2*	CGGCTGAAGTCTCCATTAGC	CCAGGGAAGTTCTGGTGTGT
*BDNF*	CAAACATCCGAGGACAAGGTGG	CTCATGGACATGTTTGCAGCATCT
*HO-1*	TCCGATGGGTCCTTACACTC	TAAGGAAGCCAGCCAAGAGA
*Nrf-2*	GCGACGGAAAGAGTATGAGC	GTTGGCAGATCCACTGGTTT
*PKC-δ*	CAACTACATGAGCCCCACCT	GAGGCTCTCTGGGTGACTTG
